# Experimental Study of Injection Molding Replicability for the Micro Embossment of the Ultrasonic Vibrator

**DOI:** 10.3390/polym14224798

**Published:** 2022-11-08

**Authors:** Tieli Zhu, Ying Liu, Tongmin Yu, Yifei Jin, Danyang Zhao

**Affiliations:** 1 School of Mechanical Engineering, Dalian University of Technology, Dalian 116024, China; 2 Mechanical Engineering Department, University of Nevada, Reno, NV 89557, USA

**Keywords:** ultrasonic vibration, injection molding, micro embossment, replicability, experimental study

## Abstract

It is challenging to fabricate micro features on an injection-molded polymer product. Ultrasonic vibration induced into micro-injection molding is helpful for flow of polymer melt. In this paper, a micro-injection mold integrated with ultrasonic vibration was designed and fabricated, and micro embossment was machined on the surface of the ultrasonic vibrator. Poly(methacrylic acid methyl ester) (PMMA) was used for injection molding experiments, with four ultrasonic power levels (0, 300, 600, and 900 W), three injection speed levels (60, 80, and 100 cm^3^/s), two injection pressure levels (60 and 90 MPa) and a mold temperature of 60 °C. It was found that ultrasonic vibration perpendicular to the middle surface of the cavity is beneficial in forming transverse microstructure, but is not conducive to generating longitudinal microstructure. Increase in injection pressure can improve molding qualities for both the longitudinal micro groove and the transverse micro groove. Increase in injection speed is not conducive to forming the longitudinal micro groove but benefits formation of the transverse micro groove. When ultrasonic vibration is applied at the injection and packing stages, molding quality of the longitudinal micro groove becomes worse, while that of the transverse micro groove becomes better.

## 1. Introduction

With rapid development of micro electro-mechanical system (MEMS) technology, polymer products with microstructural characteristics have been widely used in many fields, such as optics, clinical medicine and micro machinery. For injection molding of polymer products that have microstructures, molding quality largely depends on design of the microstructural cavity and on injection process parameters including mold temperature, polymer melt temperature, injection speed, injection pressure, and packing pressure. During micro-injection molding, fluidity of polymer melt at cavity microstructure is poor, which leads to low precision of microstructural reproduction. Increasing temperature can reduce viscosity of polymer melt, thus improving its flow capacity. When cavity temperature is raised close to the glass transition temperature of polymer, microstructural replication precision can be improved significantly [1,2]. However, high temperature of the mold inevitably delays cooling of the molded product; thus, the molding cycle is prolonged. On the other hand, the macro surface of the molded product is prone to displaying sink marks [3,4]. Application of ultrasonic vibration to micro-injection molding can promote movement of molecules, resulting in rising polymer melt temperature and reduction of polymer melt viscosity [5,6]. Appropriate ultrasonic parameters depend on specific forming conditions. Sato et al. [7] carried out ultrasonic-assisted injection molding of grating components with polystyrene (PS). Their ultrasonic vibrator had a frequency of 19 kHz, maximum power of 1200 W and maximum amplitude of 11 μm. They applied ultrasonic vibration to the injection stage and found that replicability of microstructure improved with an increase of ultrasonic vibration time. Lu et al. [8] studied effect of ultrasonic application time on weld-mark strength with polystyrene (PS) at low mold temperature (40 and 60 °C). Their experimental data indicated that application of ultrasonic vibration should be limited to the injection and packing stages, not extended to the whole molding process. Wei et al. [9] measured viscosity of low-density polyethylene (LDPE) at three ultrasonic power levels (300, 500, and 800 W), and found that the higher the ultrasonic power, the lower the viscosity of polymer melt. Xie et al. [10] investigated effects of ultrasonic application time and ultrasonic power (400, 600, and 800 W) on weld-mark strength with polystyrene (PS) at a high mold temperature (150 °C). Their results revealed that the longer that ultrasonic vibration was applied, the greater the weld-mark strength, and weld intensity was highest when ultrasonic power was 400 W. In previous studies, applied positions of ultrasonic vibration included the plasticizing unit [11,12], the runner system [13,14], the side [10], or the opposite [15] of the cavity microstructure, or ultrasonic vibration was applied to the microstructural cavity insert through threading or welding of the ultrasonic vibrator with the microstructural cavity insert [6,16].

Power and amplitude of the ultrasonic vibrator are limited by the internal space of the injection mold. In order to maximize the effect of ultrasonic vibration on formation of the microstructure, we machined the micro embossment on the surface of the ultrasonic vibrator with electric discharge machining (EDM) to directly use the ultrasonic vibrator as the microstructural cavity insert. For the first time, this work unveiled the relationships between replication of products and orientation of microstructures during ultrasonic vibration-assisted injection molding. The ultrasonic vibration field can promote unwinding of polymer chains. Moreover, cavitation and thermal effects of the ultrasonic wave can transform mechanical vibration energy into the internal energy of polymer melt so that polymer melt near the vibrating cavity cools slowly. These two aspects result in low viscosity and good flowing ability of polymer melt near the vibrating cavity.

## 2. Product Design and Molding Material

**Figure 1:**
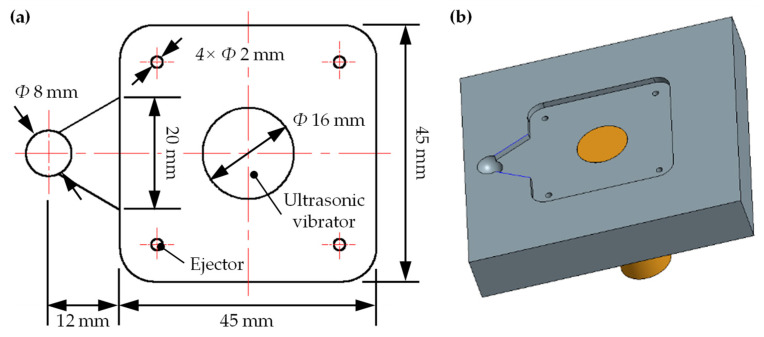
The gate and the cavity. (**a**) Planar dimensions and (**b**) three-dimensional model (including the upper part of the ultrasonic vibrator).

In order to enhance uniformity of polymer melt filling, a fan-shaped gate was used. As shown in Figure 1a, the diameter of the end of the main channel was 8 mm, and the fan gate, with a center angle of 60°, was between the end of the main channel and the cavity. The size of the polymer product was 45 mm long, 45 mm wide, and 2 mm thick. Four ejectors of *Φ* 2.0 mm were arranged at the four corners of the cavity. The ultrasonic vibrator was placed at the center of the cavity. Figure 1b shows assembly of the cavity insert and the ultrasonic vibrator.

**Figure 2:**
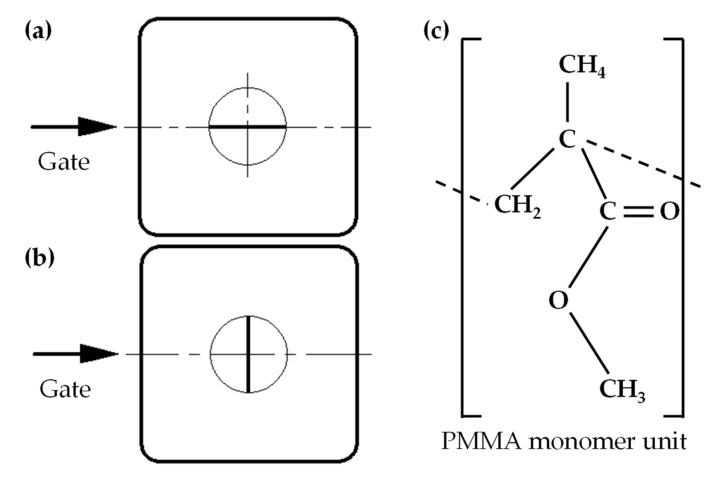
Longitudinal microstructure and transverse microstructure. (**a**) Length direction of the longitudinal microstructure parallel to the polymer-melt filling direction in the cavity and (**b**) length direction of the transverse microstructure perpendicular to the polymer-melt filling direction in the cavity. (**c**) Schematic of molecular structure of PMMA.

The upper part of the ultrasonic vibrator assembled in the cavity insert was a cylinder with a diameter of 16 mm so that micro embossment on the surface of the ultrasonic vibrator could be rotated with respect to the cavity insert. When length direction of the microstructure was parallel to the polymer-melt filling direction in the cavity, it was called longitudinal microstructure, as shown in Figure 2a. When length direction of the microstructure was perpendicular to the polymer-melt filling direction in the cavity, it was called transverse microstructure, as shown in Figure 2b.

The polymer used for injection molding was PMMA (MF001, Mitsubishi Chemical Polymer Nantong Co. LTD, Nantong, China) (Figure 2c). The selected polymer in this work had a density of 1.19 g/cm^3^, an elastic modulus of 3.33 GPa, tensile strength of 66.00 MPa, and flexural strength of 120.00 MPa. Because of its good mechanical properties and excellent transparency, PMMA has been widely used to fabricate optical products for diverse applications [17,18,19,20].

## 3. Injection Molding System and Experimental Design

**Figure 3:**
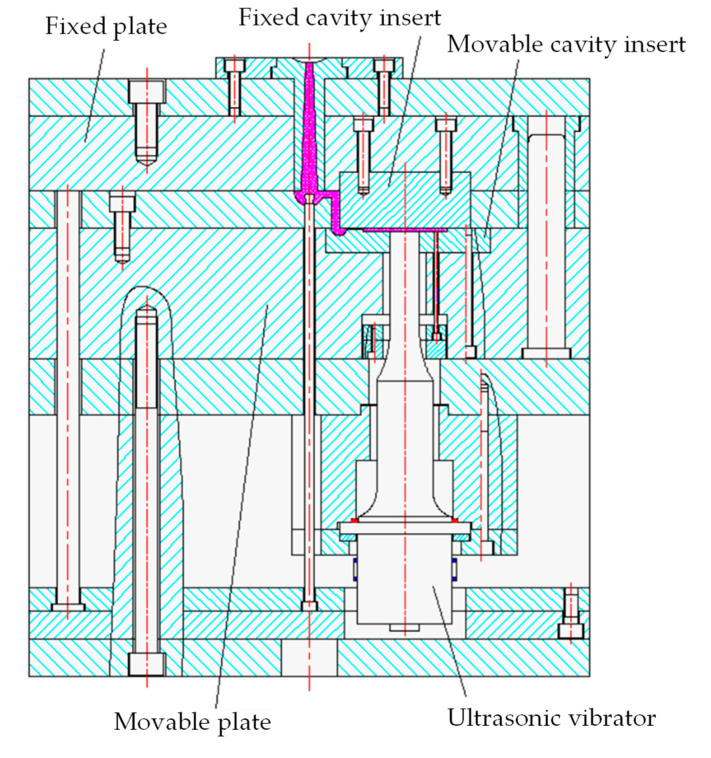
Structure of the ultrasonic-assisted injection mold.

The structure of the ultrasonic-assisted injection mold is shown in Figure 3. The cavity consisted of an ultrasonic vibrator, a movable cavity insert, and a fixed cavity insert.

**Figure 4:**
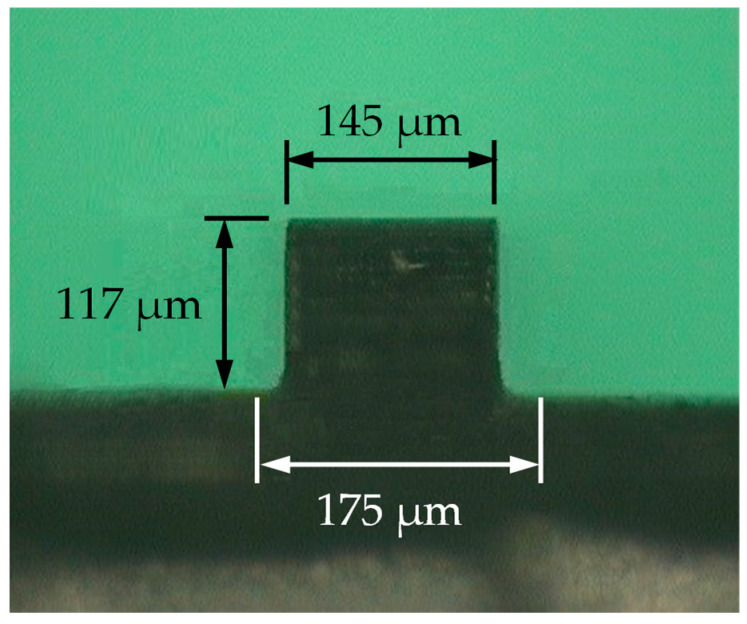
Micro embossment on the surface of the ultrasonic vibrator, machined by electrical discharge.

The ultrasonic vibrator was made of titanium alloy for the sake of strength. The micro embossment on the surface of the ultrasonic vibrator was machined by EDM. Considering the ability to resist arc ablation, the material of the tool electrode was copper–tungsten alloy with 75% tungsten content [21,22]. The cross-section size of the machined micro embossment (117 μm in height and 145 μm in width at the top and 175 μm in width at the root) is shown in Figure 4.

The PMMA was dried before injection molding due to high moisture absorption. Drying temperature was 85 °C and drying time was 4 h. Processing parameters of injection molding experiments are illustrated in Table 1. Injection pressure had two levels (60 and 90 MPa). Injection speed had three levels (60, 80, and 100 cm^3^/s). Ultrasonic power had four levels (0, 300, 600, and 1000 W). Constant processing parameters included a PMMA melt temperature of 240 °C, mold temperature of 60 °C, packing pressure of 20 MPa, packing time of 5s, and ultrasonic time (starting from injecting moment) of 4 s. Ultrasonic frequency was 21 kHz. All quantitative values in the text and figures were reported as means ± standard deviation (SD), with *n* = 5 samples per group.

In micro-injection molding, high mold temperature (approaching the glass transition temperature of polymer) is more likely to be adopted for the sake of improving flow capacity of polymer melt [23,24,25,26]. However, high mold temperature is often accompanied by sink marks and prolonged molding cycle [27,28,29,30,31]. Therefore, we used a low mold temperature of 60 °C with the premise of fully filling the cavity.

## 4. Measuring Instruments and Method

Every time processing parameters were changed in the experiments and the first five plastic parts were discarded. Then, the injection molding machine was considered to work stably, and the next five plastic parts were taken as experimental samples.

**Figure 5:**
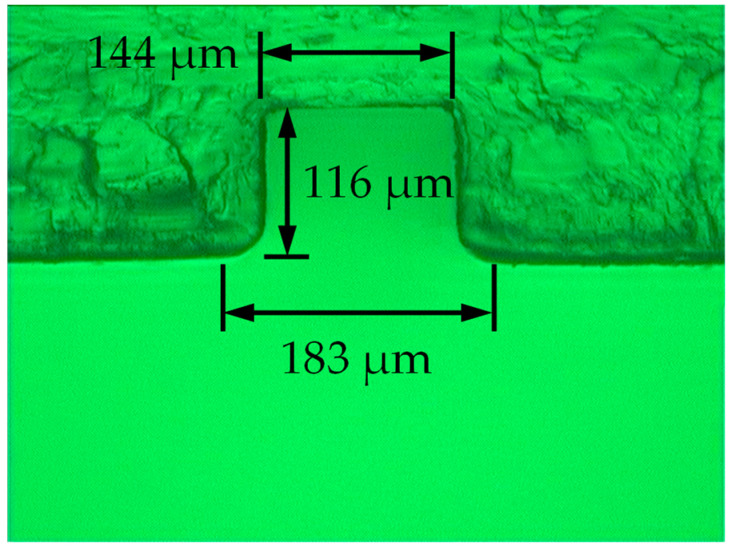
An image of the plastic transverse micro groove molded by injection pressure of 60 MPa, injection speed of 100 cm^3^/s, and ultrasonic power of 600 W.

The measuring position was selected at the midpoint of the length of the micro groove. Polymer products obtained by different molding parameters were sliced. Then, cross-section photos of the micro grooves were taken by a universal tool microscope. It was found that the rounded corner at the opening of the molded micro groove was larger than the rounded corner at the root of the micro embossment of the mold cavity because the root of the cavity micro embossment was difficult to fill completely. That is to say, the opening width of the molded micro groove was larger than the root width of the micro embossment of the cavity. In addition, opening width of the molded micro groove varied greatly with processing parameters, while depth and bottom width of the molded micro groove were more stable and rarely affected by processing parameters. Therefore, influence of processing parameters on opening width of the micro groove is mainly discussed. Figure 5 is an example of a micro groove detection image of a polymer product slice.

## 5. Experimental Results and Discussion

### 5.1. Opening Width of Longitudinal Micro Groove

**Figure 6:**
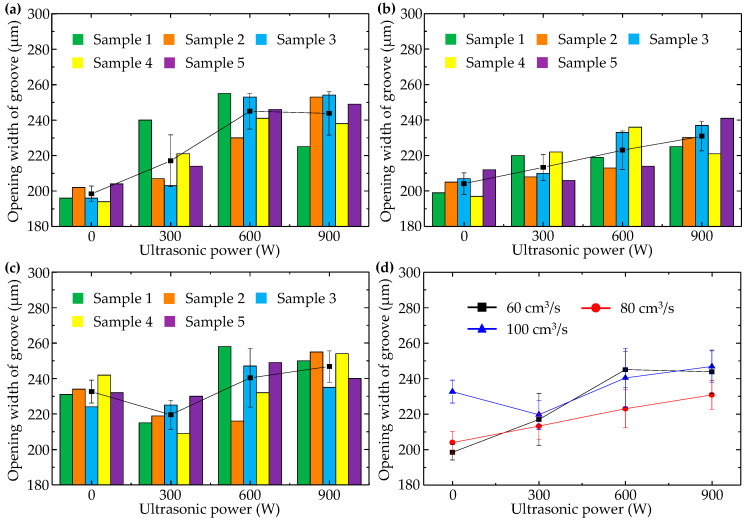
Variation of opening width of the longitudinal micro groove with ultrasonic power when injection pressure was 60 MPa and injection speeds were (**a**) 60 cm^3^/s, (**b**) 80 cm^3^/s, and (**c**) 100 cm^3^/s. (**d**) Comparison of the average values of each group.

When injection pressure was 60 MPa, changes of longitudinal micro groove opening width with injection speed and ultrasonic power were illustrated in Figure 6. Figure 6a–c separately show sample data corresponding to injection speeds of 60, 80, and 100 cm^3^/s. Comparison of the average values of each group of sample data is shown in Figure 6d.

It can be seen that with rise in injection speed, opening width of the longitudinal micro groove increased, which meant falling replicability and worse molding quality. This phenomenon can be explained by the fact that when injection pressure is low, high injection speed only urges polymer melt to rapidly spread forward in the direction of inertial motion, and the “detail” of filling the longitudinal micro embossment of the cavity is omitted. When polymer melt reached the end of the flow path and completed filling the macro area of the cavity, cavity pressure peaked but was insufficient to drive polymer melt into the space around the longitudinal embossment of the cavity because polymer melt viscosity increased sharply as temperature dropped.

In addition, with rising ultrasonic power, opening width of the longitudinal micro groove increased in general. In Figure 6d, minimum opening width (198 μm) of the longitudinal micro groove appeared when injection speed was 60 cm^3^/s in the absence of ultrasound. This shows that ultrasonic vibration has a negative effect on formation quality of the longitudinal micro groove. Because orientation of macromolecules usually coincided with flow direction of polymer melt, macromolecules were oriented along length direction of the longitudinal micro groove, i.e., perpendicular to the cross-section of the longitudinal micro groove. Thus, for the longitudinal micro groove, ultrasonic vibration perpendicular to the middle surface of the cavity had no effect on the orientation state of macromolecules. Not only that, but polymer chains that were arranged along the length direction of the longitudinal micro groove were pushed away from the micro embossment of the cavity by ultrasonic vibration perpendicular to the middle surface of the cavity, resulting in increased opening width of the molded longitudinal micro groove. There was one exception: when injection speed was 100 cm^3^/s. In that case, because high-speed filling without ultrasound led to excessive opening width of the longitudinal micro groove, there was an obvious reduction of micro groove opening width at 300 W ultrasonic power. This indicates that ultrasonic vibration of low power may affect the stacking state of polymer chains that are oriented along the length direction of the longitudinal micro groove to a certain extent and make macromolecules move closer to the root of the cavity micro embossment.

**Figure 7:**
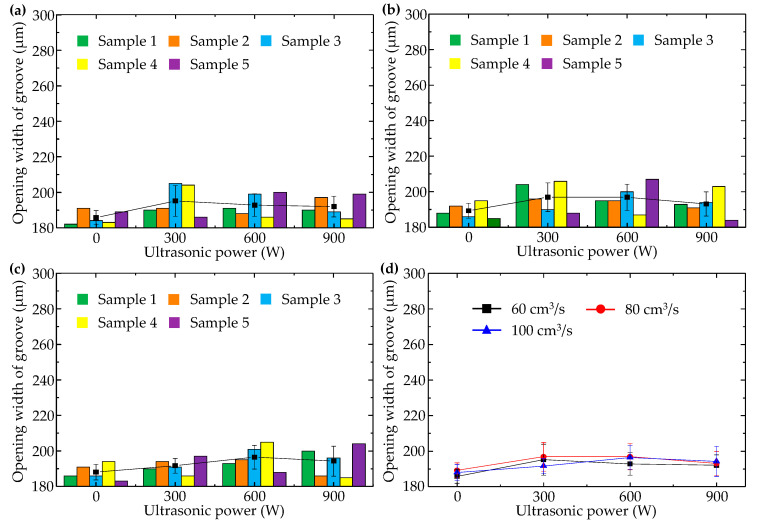
Variation of opening width of the longitudinal micro groove with ultrasonic power when injection pressure was 90 MPa and injection speeds were (**a**) 60 cm^3^/s, (**b**) 80 cm^3^/s, and (**c**) 100 cm^3^/s. (**d**) Comparison of the average values of each group.

When injection pressure was 90 MPa, changes of longitudinal micro groove opening width with injection speed and ultrasonic power were illustrated in Figure 7. In comparison with Figure 6, it can be seen that longitudinal micro groove opening width could be significantly reduced by increasing injection pressure, whether or not ultrasonic vibration was applied. With high pressure (90 MPa), change of injection speed had no obvious effect on opening width of the longitudinal micro groove, illustrating that the promoting effect of high pressure surpassed the adverse effect of high speed on longitudinal micro groove formation and played a dominant role.

Moreover, ultrasonic vibration still enlarged the opening width of the longitudinal micro groove, but enlargement was not as dramatic as when injection pressure was lower (60 MPa). In Figure 7d, minimum opening width (186 μm) of the longitudinal micro groove appeared when injection speed was 60 cm^3^/s in the absence of ultrasound. The minimum value (182 μm) of sample data of longitudinal micro groove opening width was as obtained in Figure 7a, under conditions of injection pressure of 90 MPa, injection speed of 60 cm^3^/s, and ultrasonic power of 0 W. Figure 7 illustrates again that ultrasonic vibration perpendicular to the middle surface of the cavity was not conducive to formation quality of the longitudinal micro groove.

### 5.2. Opening Width of Transverse Micro Groove

**Figure 8:**
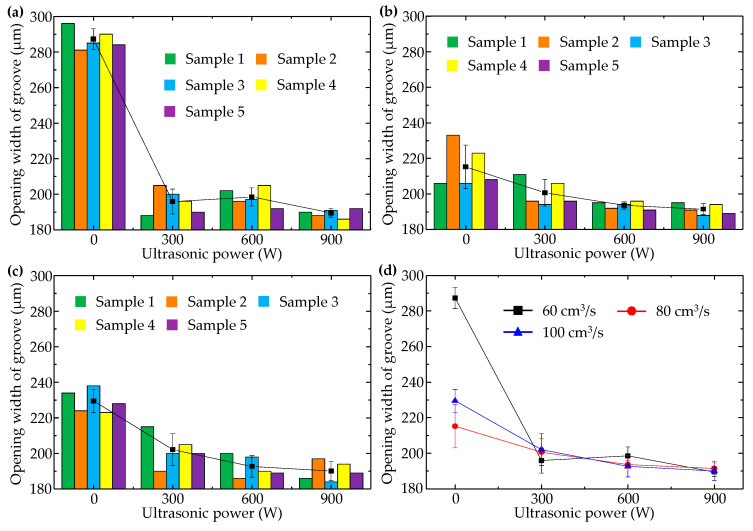
Variation of opening width of the transverse micro groove with ultrasonic power when injection pressure was 60 MPa and injection speeds were (**a**) 60 cm^3^/s, (**b**) 80 cm^3^/s, and (**c**) 100 cm^3^/s. (**d**) Comparison of the average values of each group.

When injection pressure was 60 MPa, changes in transverse micro groove opening width with injection speed and ultrasonic power were shown in Figure 8. In comparison with Figure 6, it can be seen that in the absence of ultrasonic vibration, formation quality of the transverse micro groove was not as good as that of the longitudinal micro groove. Especially during filling of the cavity at low speed (60 cm^3^/s), opening width of the transverse micro groove was nearly 90 μm larger than that of the longitudinal micro groove, and increasing injection speed could reduce opening width of the transverse micro groove. This phenomenon can be explained by the fact that no two sides of the transverse micro groove were formed at the same time.

**Figure 9:**
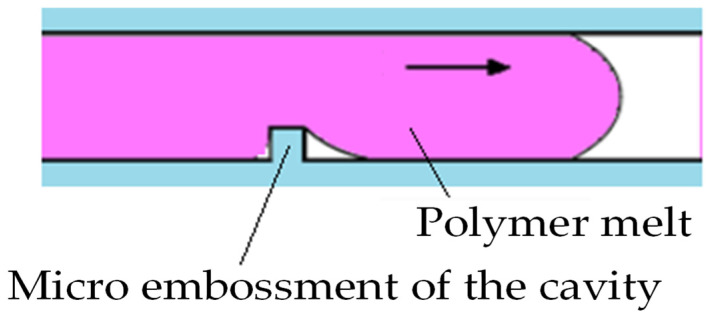
Filling of the transverse micro embossment of the cavity.

As shown in Figure 9, in the cavity-filling process, the side of the transverse micro embossment close to the gate was filled first, and after getting over the transverse micro embossment of the cavity, polymer melt followed the original flow direction instead of filling the other side of the transverse micro embossment away from the gate immediately. When filling of the cavity macro area was completed, polymer melt was driven by the sudden increased cavity pressure into the space around the other side of the transverse micro embossment, away from the gate. The higher the injection rate, the faster the polymer melt returned to fill the other side of the transverse micro embossment away from the gate, and the better the polymer melt fluidity.

It can also be seen in Figure 8 that ultrasonic vibration had a significant effect on improving molding quality of the transverse micro groove. After ultrasonic vibration was applied to the injection and packing stages, opening width of the transverse micro groove decreased to about 200 μm. Especially when ultrasonic power was 900 W, opening width of the transverse micro groove was nearly 190 μm: even smaller than the minimum value (198 μm) of the longitudinal micro groove opening width in Figure 6d when injection pressure was the same as 60 MPa. The reason why ultrasonic vibration reduces opening width of the transverse micro groove is that ultrasonic vibration changes the orientation state of polymer molecules. Macromolecular chains are originally oriented along the flow direction of polymer melt. However, ultrasonic vibration perpendicular to the middle surface of the cavity causes macromolecular chains to bend in order to fit the profile of the cavity transverse to micro embossment. Formation quality of the transverse micro groove is thus improved.

Moreover, ultrasonic vibration makes the effect of injection speed on opening width of the transverse micro groove become weak. As shown in Figure 8d, corresponding points of the three curves with injection speeds of 60, 80, and 100 cm^3^/s almost coincided with each other when ultrasonic power was 900 W. This indicates that the promoting effect of ultrasonic vibration surpasses the promoting effect of high speed on micro transverse groove formation.

**Figure 10:**
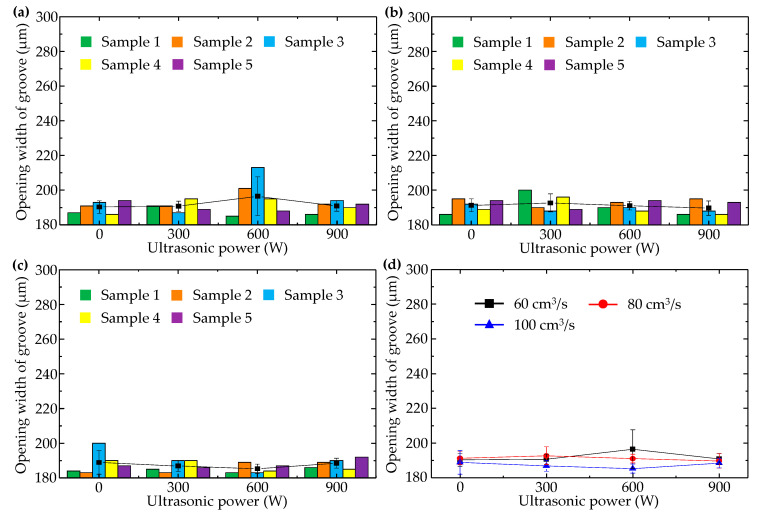
Variation of opening width of the transverse micro groove with ultrasonic power when injection pressure was 90 MPa and injection speeds were (**a**) 60 cm^3^/s, (**b**) 80 cm^3^/s, and (**c**) 100 cm^3^/s. (**d**) Comparison of the average values of each group.

When injection pressure was 90 MPa, changes in transverse micro groove opening width with injection speed and ultrasonic power were shown in Figure 10. In comparison with Figure 8, it can be seen that in the absence of ultrasonic vibration, transverse micro groove opening width could be significantly reduced with increasing injection pressure. In addition, with high pressure (90 MPa), change of injection speed had no obvious effect on opening width of the transverse micro groove, again illustrating that the promoting effect of high pressure on transverse micro groove formation played a dominant role. Moreover, effect of ultrasonic vibration on opening width of the transverse micro groove was not as dramatic as when injection pressure was lower (60 MPa). In Figure 10d, minimum opening width (185 μm) of the transverse micro groove appeared when injection speed was 100 cm^3^/s and ultrasonic power was 600 W. The minimum value (183 μm) of sample data of transverse micro groove opening width was as obtained in Figure 10c under conditions of injection pressure of 90 MPa, injection speed of 100 cm^3^/s, and ultrasonic power of 600 W.

## 6. Conclusions

The ultrasonic-assisted micro-injection mold designed in this paper took the surface of the ultrasonic vibrator as a component of the microstructural cavity. A micro embossment with a cross-section size of 117 μm high and 145 μm wide at the top and 175 μm wide at the root was machined on the surface of the ultrasonic vibrator. Improvement measures for formation quality of the longitudinal micro groove parallel to the filling direction and the transverse micro groove perpendicular to the filling direction were discussed. A comprehensive comparison of different injection molding experiments showed that ultrasonic vibration perpendicular to the middle surface of the cavity is beneficial to formation of the transverse micro groove, but, however, unfavorable to formation of the longitudinal micro groove. If ultrasonic vibration is used to improve molding quality of micro grooves, the gate should be arranged to make the filling direction of polymer melt not parallel to the length direction of the micro groove; that is to say, the micro groove of the polymer product should avoid the position of the longitudinal micro groove.

Without raising mold temperature close to the glass transition temperature of polymer, formation quality of the longitudinal micro groove was better than that of the transverse micro groove when ultrasonic vibration was not applied. The way to improve molding quality of the longitudinal micro groove is to increase injection pressure. The ways to improve molding quality of the transverse micro groove are (1) to increase injection speed when injection pressure is low, (2) to introduce ultrasonic vibration when injection pressure is low, and (3) to increase injection pressure.

The longitudinal micro groove obtained a minimum opening width of 182 μm under conditions including ultrasonic power of 0 W, injection speed of 60 cm^3^/s, injection pressure of 90 MPa, and mold temperature of 60 °C. The transverse micro groove obtained a minimum opening width of 183 μm under conditions including ultrasonic power of 600 W, injection speed of 100 cm^3^/s, injection pressure of 90 MPa, and mold temperature of 60 °C.

## Figures and Tables

**Table 1:** Injection molding process parameters.

Process Parameters	Values
Injection pressure (MPa)	60, 90
Injection speed (cm^3^/s)	60, 80, 100
Ultrasonic power (W)	0, 300, 600, 900
PMMA melt temperature (°C)	240
Mold temperature (°C)	60
Packing pressure (MPa)	20
Packing time (s)	5
Ultrasonic time (s)	4

## Data Availability

The data that support the findings of this study are available from the corresponding author (yifeij@unr.edu) upon reasonable request.
